# The burden and challenges of tuberculosis in China: findings from the Global Burden of Disease Study 2015

**DOI:** 10.1038/s41598-017-15024-1

**Published:** 2017-11-03

**Authors:** Sui Zhu, Lan Xia, Shicheng Yu, Saobing Chen, Juying Zhang

**Affiliations:** 1 Department of Epidemiology and Biostatistics, West China School of Public Health, Sichuan University, Sichuan, 610044 China; 2 Sichuan Provincial Center for Disease Control and Prevention, Chengdu, P.R. China; 3 Office of Epidemiology, Chinese Center for Disease Control and Prevention, Beijing, P.R. China

## Abstract

To achieve the End Tuberculosis (TB) Strategy, it is important to understand the characteristics of TB in China, which may provide the government with important clues for controlling TB by 2030. Data from the Global Burden of Disease Study 2015 (GBD 2015) and Institute for Health Metrics and Evaluation (IHME) were reviewed and analysed. The age-standardized death rate decreased by 83.79% [95% uncertainty interval (UI) 73.06–87.10] from 1990 to 2015. The age-standardized prevalence of TB in males decreased steadily by 33.88% (95% UI 29.35–37.67) but nearly increased by 6.24% (95% UI -2.02–15.07) in females from 1990 to 2015. Disability-adjusted life years (DALYs) were higher in males than in females, and the highest TB burden was found in the elderly (70+ years of age). Over the period 1990–2015, the attributable age-standardized DALY rates for smoking decreased by 12.98% (95% UI 2.40–24.27), but increased for alcohol use and high fasting plasma glucose (HFPG). Greater attention should be paid to females especially in the under 5 years of age group, and more latent reasons explaining TB DALYs should be explored in future studies.

## Introduction

Tuberculosis (TB) is caused mainly by Mycobacterium tuberculosis (*M*.*tb*), and pulmonary TB is typical and infectious^1^. Although only a small proportion (5–10%) of people who are infected with *M*.*tb* will develop the active TB during their lifetime, TB is one of the top 10 causes of death worldwide^2^. From the Global Burden of Disease Study 2015 (GBD 2015), TB ranked only below human immunodeficiency virus (HIV)/acquired immune deficiency syndrome (AIDS) in 2015, with 10.18 million new TB cases and 1.32 million deaths at all ages globally^3^. Moreover, people living with HIV accounted for 13% (1.35 million) of all new TB cases and 15% (0.21 million) of the TB deaths in 2015^4^.

Fortunately, TB is curable and preventable. Significant progress in TB control has been achieved worldwide, and the age-standardized mortality rate of TB declined by 33.88% from 2005 to 2015. However, the global burden of TB remains high^3^. The world health organization (WHO) established an ambitious End TB Strategy, which outlines a 90% reduction in TB deaths and an 80% reduction in the TB incidence rate in 15 years starting in 2015^5^. Additionally, the Strategy calls for reducing the TB death and incidence rates by 35% and 20%, respectively, by 2020. The End TB Strategy is fully aligned with the framework of the Sustainable Development Goals (SDGs) and combines a holistic mix of health and social interventions.

According to the GBD 2015, 15.32% of the new TB cases (1.56 million) and 3.90% of the TB deaths (51.52 thousand) were reported in China^4^, which was one of the 30 highest TB burden countries worldwide, ranking only below India and Indonesia in 2015^2^. Thus, to achieve the End TB Strategy, greater attention should be given to China in the period 2017–2030. China is the world’s third-largest country in area, with a population of 1.37 billion and a gross domestic product (GDP) per capital of 49,992 Renminbi (RMB) in 2015^6^. During the past several decades of steady economic growth, enormous strides in improving health have been made in China. Life expectancy at birth has increased by 9.60 years, and the burden of all causes disability-adjusted life years (DALYs) declined by 19.15% from 1990 to 2015^7^. The Directly Observed Treatment, Short-Course (DOTS) strategy was introduced in 13 provinces, covering 50% of the Chinese population in 1991, and expanded nation-wide to cover 100% of the population in 2005^8,9^.

Through these efforts, China achieved the Millennium Development Goal of a 50% reduction in TB prevalence and mortality in 2015. However, the incidences of HIV with TB, chronic disease with TB and multi-drug resistant TB (MDR-TB) have all increased in recent years^10–13^, which may hamper TB control strategies. One study indicated that China could not achieve the global target of the End TB Strategy by 2035 if it were to maintain the current DOTS strategy by using an individual-based computational model of TB transmission, with a declining incidence and mortality of 42% and 41%, respectively^14^.

Therefore, this study aims to provide important clues for the government to achieve its End TB strategy targets, by comprehensively overviewing TB trends and changes in mortality, prevalence and DALYs according to GBD 2015 and the Institute for Health Metrics and Evaluation (IHME) website^4^.

## Results

A total of 2.49 million TB deaths were reported from 1990 to 2015 in China, and 67.07% were males (1.67 million). The TB prevalence and DALYs were estimated every 5 years from 1990 to 2015, and a total of 10.38 million TB cases (6.29 million for males) and 20.68 million DALYs (13.50 million for males) were reported from 1990 to 2015 in China. In this study, the means and 95% uncertainty intervals (UIs) and median of the rate changes were determined for the period between 1990 and 2015.

### TB Mortality

**Figure 1:**
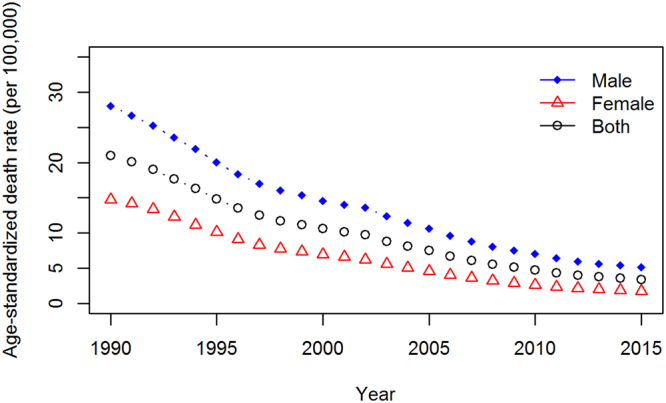
Age-standardized death rates of TB decreased from 1990 to 2015 for both sexes in China. The age-standardized death rates decreased annually, and the male age-standardized death rates were always higher than were the female rates from 1990 to 2015.

In 2015, there were an estimated 48,922 TB deaths in China, of which 35,936 (73.46%) were among men and 12,985 (26.54%) among women. The total number of deaths from TB significantly declined by 69.34% (95% UI: 51.38–75.31) from 1990 (159,592) to 2015 (48,922) for both sexes. The age-standardized death rates decreased rapidly by 83.79% (95% UI 73.06–87.10) from 1990 to 2015. The age-standardized death rates steadily decreased annually, from 20.06 (95% UI 18.47–40.85) to 3.40 (95% UI 2.86–5.37) per 100,000 persons from 1990 to 2015 for both sexes (Fig. 1). The age-standardized death rates were always higher in males than in females, but the difference between males and females decreased from 13.33 to 3.33 per 100,000 persons from 1990 to 2015. The age-specific death rates increased continuously with age, and the highest death rate was observed in the elderly (70+ years of age) from 1990 to 2015 for both sexes (Supplementary Table S1).

### Prevalence of TB

Although the prevalence of TB changed little from 134.27 (95% UI 119.32–150.96) in 1990 to 137.93 (95% UI 124.93–151.07) in 2015 per 100,000 persons, the age-standardized prevalence fell by 20.65% (95% UI 15.07–25.65) from 1990 to 2015. The epidemiologic situations with all-age and age-standardized prevalence of TB were different between males and females from 1990 to 2015, with a significant decreasing of 11.78% (95% UI 5.86–17.03) and 33.88% (95% UI 29.35–37.67) in males, respectively, but an significant increase of 29.93% (95% UI 19.52–40.88) in all-age and nearly increase of 6.24% (95% UI −2.02–15.07) in age-standardized in females. A similar difference was found for the under-5 population. The under-5 prevalence for males decreased by 32.28% (95% UI 16–44.89) from 1990 to 2015. However for females, the under-5 prevalence increased by 189.19% (95% UI 141.71–266.56), from 8.20 (95% UI 5.45–11.46) per 100,000 persons in 1990 to 23.71 (95% UI 18.51–29.85) per 100,000 persons in 2015 (Table 1).

**Table 1:** Prevalence of TB by age-sex from 1990 to 2015 in China (per 100,000 persons, 95% UI).

Age-sex	1990	1995	2000	2005	2010	2015	%$\bigtriangleup$ (1990–2015)
**Both Sexes**							
**under 5**	**11**.**95** (**8**.**48**–**15**.**82**)	**19**.**73** (**15**.**11**–**24**.**93**)	**18**.**16** (**13**.**67**–**23**.**35**)	**19**.**74** (**15**.**37**–**25**.**00**)	**10**.**68** (**7**.**74**–**14**.**14**)	**16**.**59** (**12**.**53**–**21**.**46**)	**38**.**79**
5–14 years	26.61 (18.30–36.86)	36.15 (27.51–46.87)	35.54 (26.40–47.46)	31.85 (24.57–41.34)	15.62(11.56–21.22)	21.32 (16.59–27.77)	−19.88
15–49 years	131.67 (107.65–153.23)	146.18 (123.73–167.54)	133.24 (112.11–153.55)	121.45 (105.77–138.10)	111.00 (96.41–128.41)	127.53 (110.03–148.72)	−3.15
50–69 years	301.64 (242.92–359.20)	238.26 (198.71–280.26)	250.89 (210.29–292.82)	201.55 (171.23–232.78)	214.72 (178.66–249.43)	186.30 (156.16–219.48)	−38.24
70+ years	527.18 (394.48–703.87)	407.85 (321.25–520.51)	477.71 (376.89–605.82)	402.90 (326.62–495.67)	454.58 (370.56–557.25)	396.88 (325.05–483.5)	−24.72
All ages	134.27 (119.32–150.96)	135.14 (121.60–149.02)	137.99 (123.82–151.52)	129.63 (118.51–140.86)	132.36 (119.90–144.80)	137.93 (124.93–151.07)	2.72
Age–standardized	158.56 (140.74–177.66)	146.80 (133.10–161.09)	147.51 (133.04–162.13)	132.61 (121.53–144.11)	126.80 (115.23–138.34)	125.82 (114.52–137.74)	−20.65
**Male**							
under 5	15.40 (11.10–20.22)	19.37 (14.70–24.52)	20.05 (15.18–25.46)	13.68 (10.10–17.99)	9.47 (6.77–12.67)	10.43 (7.36–14.22)	−32.28
5–14 years	23.46 (16.21–32.82)	25.31 (18.32–33.81)	32.03 (23.31–42.82)	18.14 (12.81–25.32)	12.94 (9.19–18.02)	14.71 (10.54–20.21)	−37.30
15–49 years	159.93 (131.20–186.06)	150.49 (125.86–174.58)	158.31 (132.28–182.83)	119.81 (101.37–137.77)	131.24 (112.73–152.58)	124.79 (106.05–147.06)	−21.97
50–69 years	422.31 (340.01–503.44)	306.53 (253.22–364.49)	353.97 (295.28–415.25)	259.77 (216.78–304.21)	318.74 (263.43–373.95)	238.04 (196.63–284.00)	−43.63
70+ years	796.31 (595.53–1067.30)	547.95 (420.83–717.42)	724.08 (565.22–926.97)	544.71 (432.74–687.97)	664.24 (535.60–820.17)	517.94 (415.10–648.67)	−34.96
All ages	170.88 (151.94–191.7)	147.11 (131.39–163.34)	172.84 (154.94–190.12)	140.18 (126.53–153.88)	171.14 (154.60–187.12)	150.76 (135.27–166.12)	−11.78
Age-standardized	211.35 (187.18–236.94)	168.05 (150.64–186.01)	192.31 (172.00–212.23)	148.59 (134.61–163.53)	168.01 (151.82–184.27)	139.75 (126.14–154.21)	−33.88
**Female**							
**under 5**	**8**.**20** (**5**.**45**–**11**.**46**)	**20**.**14** (**15**.**40**–**25**.**64**)	**16**.**01** (**11**.**74**–**21**.**09**)	**26**.**75** (**21**.**27**–**32**.**92**)	**12**.**09** (**8**.**95**–**15**.**85**)	**23**.**71** (**18**.**51**–**29**.**85**)	**189**.**19**
5–14 years	29.94 (20.52–41.94)	47.77 (37.25–60.76)	39.37 (29.69–52.18)	47.22 (37.53–59.24)	18.68 (14.18–24.89)	28.99 (23.16–36.47)	−3.20
15–49 years	101.81 (83.34–119.15)	141.64 (122.40–162.51)	106.90 (90.54–123.14)	123.18 (109.26–139.37)	89.55 (77.94–104.07)	130.45 (113.84–150.12)	28.13
50–69 years	171.12 (137.53–203.59)	164.25 (139.50–189.07)	141.47 (118.71–164.17)	140.57 (122.77–159.6)	107.26 (91.23–124.09)	133.40 (115.10–153.55)	−22.04
70+ years	314.59 (237.57–417.41)	294.49 (239.57–361.88)	272.90 (220.34–339.80)	280.64 (235.11–331.06)	269.19 (223.10–323.82)	289.04 (244.68–341.13)	−8.12
All ages	95.68 (84.76–107.88)	122.50 (110.83–134.17)	101.19 (90.83–111.45)	118.47 (109.37–128.69)	91.27 (83.33–100.43)	124.31 (113.55–136.07)	29.93
Age-standardized	106.62 (94.73–119.14)	126.21 (115.24–137.09)	103.43 (93.28–113.32)	118.10 (109.18–127.49)	86.31 (78.94–94.46)	113.28 (103.98–123.33)	**6**.**24**

TB: tuberculosis; UI: uncertainty intervals; %$\bigtriangleup$: the percent changes of death rate from 1990 to 2015.

### DALYs of TB

**Figure 2:**
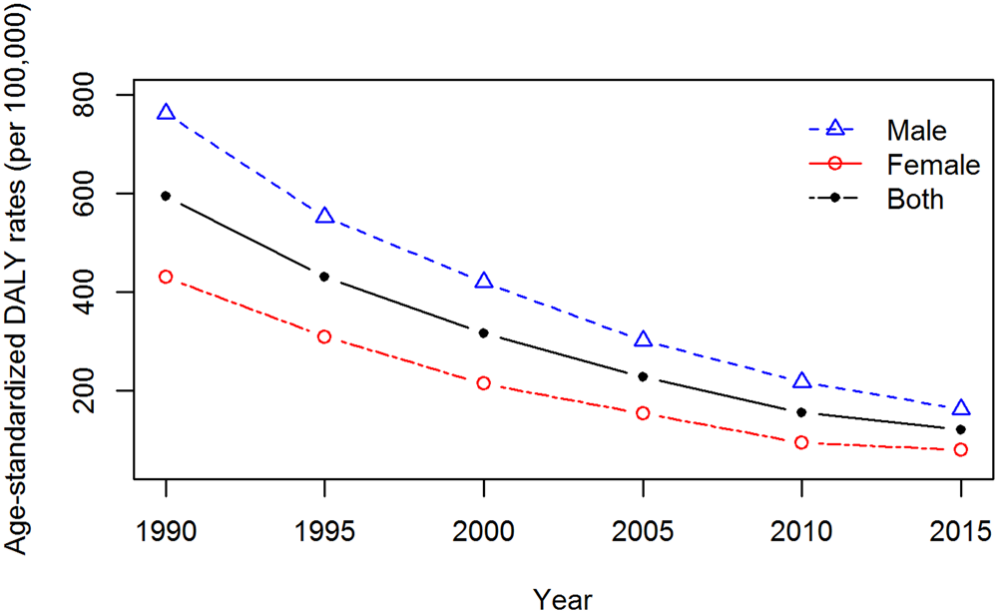
Age-standardized DALYs of TB decreased from 1990 to 2015 for both sexes in China. The burden of TB in males was much higher than in females, and the total DALYs of TB decreased from 5.59 million (95% UI 4.31–6.68) in 1990 to 1.84 million (95% UI 1.54–2.48) in 2015.

The total DALYs due to TB decreased from 5.59 million (95% UI 4.31–6.68) in 1990 to 1.84 million (95% UI 1.54–2.48) in 2015. From 1990 to 2015, the reductions in the age-standardized DALY rate was 79.85% (95% UI 72.53–83.12). Similar to the death rates and prevalence, the TB burden was much higher for males than for females at all time points (Fig. 2). Furthermore, TB burdens in the elderly (70+ years of age) was much higher than those in the other groups from 1990 to 2015 (Table 2).

**Table 2:** Different changes of age-standardized DALYs of TB from 1990 to 2015 for both sexes in China (per 100,000 persons, 95% UI).

Age-sex	1990	1995	2000	2005	2010	2015	%$\bigtriangleup$ (1990–2015)
**Both Sexes**							
under 5	226.06 (125.48–305.31)	144.16 (96.66–204.81)	117.43 (76.61–146.38)	70.18 (47.15–85.09)	34.41 (26.12–49.33)	23.93 (18.53–36.47)	−89.41
5–14 years	106.48 (77.12–125.10)	74.26 (59.69–90.07)	57.94 (46.34–69.82)	37.63 (31.09–44.41)	25.65 (20.92–31.61)	18.83 (15.20–23.40)	−82.32
15–49 years	400.21 (318.57–474.33)	329.59 (268.49–402.6)	240.99 (196.86–292.49)	173.02 (142.29–209.36)	121.67 (104.04–154.52)	101.73 (83.96–130.24)	−74.58
50–69 years	1245.35 (893.39–1502.07)	858.11 (658.91–1073.14)	622.89 (507.05–786.15)	432.09 (360.69–562.27)	292.00 (248.73–405.84)	223.23 (185.85–316.87)	−82.08
70+ years	1909.56 (1361.33––2483)	1356.15 (1052.12–1754.68)	1032.87 (851.59–1323.36)	758.88 (646.81–992.51)	544.03 (463.06–771.51)	407.13 (338.84–581.73)	−78.68
All ages	484.41 (373.22–578.31)	368.76 (298.70–450.57)	284.66 (232.45–351.29)	219.4 (183.05–275.12)	160.75 (137.59–213.61)	132.87 (111.58–179.43)	−72.57
Age-standardized	593.63 (452.72–708.57)	429.56 (346.8–525.08)	316.1 (260.16–390.48)	226.7 (190.27–287.24)	154.63 (132.71–205.92)	119.60 (100.75–161.19)	−79.85
**Male**							
under 5	323.25 (188.32–508.55)	215.86 (144.21–329.8)	189.72 (114.90–237.16)	109.69 (67.36–135.97)	51.04 (38.09–80.38)	31.28 (23.62–56.10)	−90.32
5–14 years	102.86 (66.41–132.73)	73.17 (49.54–98.33)	63.1 (43.05–81.52)	34.83 (24.68–44)	24.96 (19.24–33.6)	16.70 (12.94–23.25)	−83.77
15–49 years	456.49 (338.95–612.27)	380.71 (293.18–514.29)	293.82 (226.73–394.76)	211.01 (162.13–278.12)	158.4 (129.03–211.12)	125.36 (101.27–174.32)	−72.54
50–69 years	1652.79 (1112.22–2226.58)	1151.57 (814.35–1602.86)	846.21 (639.91–1172.89)	593.37 (452.81–822.46)	429.12 (358.31–630.38)	323.11 (264.94–486.62)	−80.45
70+ years	2754.40 (1927.89–3942.49)	1927.86 (1414.95–2765.99)	1516.86 (1173.13–2141.65)	1117.2 (870.00–1581.4)	826.98 (696.78–1216.41)	606.16 (496.62–920.10)	−77.99
All ages	595.17 (420.98–798.94)	454.65 (341.32–624.63)	364.78 (282.12–493.39)	282.22 (218.49–379.15)	219.79 (183.54–310.53)	176.02 (145.81–253.69)	−70.43
Age-standardized	761.77 (534.90–1040.61)	551.30 (408.61–763.44)	419.48 (322.92–568.66)	301.13 (235.08–406.78)	216.62 (181.16–307.46)	161.33 (133.79–229.99)	−78.82
**Female**							
under 5	120.41 (42.19–166.37)	64.15 (36.3–79.69)	35.30 (27.49–48.58)	24.49 (19.09–30.9)	15.04 (10.22–18.72)	15.42 (10.54–20.46)	−87.19
5–14 years	110.31 (78.53–134.81)	75.43 (58.70–96.41)	52.29 (42.44–68.94)	40.77 (32.78–50.15)	26.44 (20.78–32.18)	21.30 (16.64–27.36)	−80.69
15–49 years	340.74 (243.53–405.98)	275.84 (213.66–327.32)	185.51 (144.46–220.53)	132.98 (107.10–156.47)	82.75 (68.60–102.16)	76.48 (60.69–98.18)	−77.56
50–69 years	804.62 (521.66–952.58)	539.96 (377.28–617.46)	385.84 (284.53–446.49)	263.12 (208.50–323.06)	150.34 (126.55–216.97)	121.12 (95.88–175.91)	−84.95
70+ years	1242.18 (766.95–1557.48)	893.52 (610.21–1072.47)	630.53 (484.00–778.86)	449.94 (387.50–600.2)	293.82 (243.46–433.41)	229.83 (179.43–334.30)	−81.50
All ages	367.65 (265.29–422.36)	278.09 (211.38–322.40)	200.06 (156.3–237.72)	152.96 (125.45–185.56)	98.18 (82.99–130.64)	87.07 (70.24–115.70)	−76.32
Age-standardized	429.72 (301.27–493.64)	308.90 (233.91–352.46)	213.32 (166.34–252.02)	153.01 (125.58–186.41)	93.17 (79.01–124.14)	78.54 (63.43–102.84)	−81.72

DALYs: disability-adjusted life years; TB: tuberculosis; UI: uncertainty intervals; %$\bigtriangleup$: the percent changes of death rate from 1990 to 2015.

### Risk factors for DALYs

**Figure 3:**
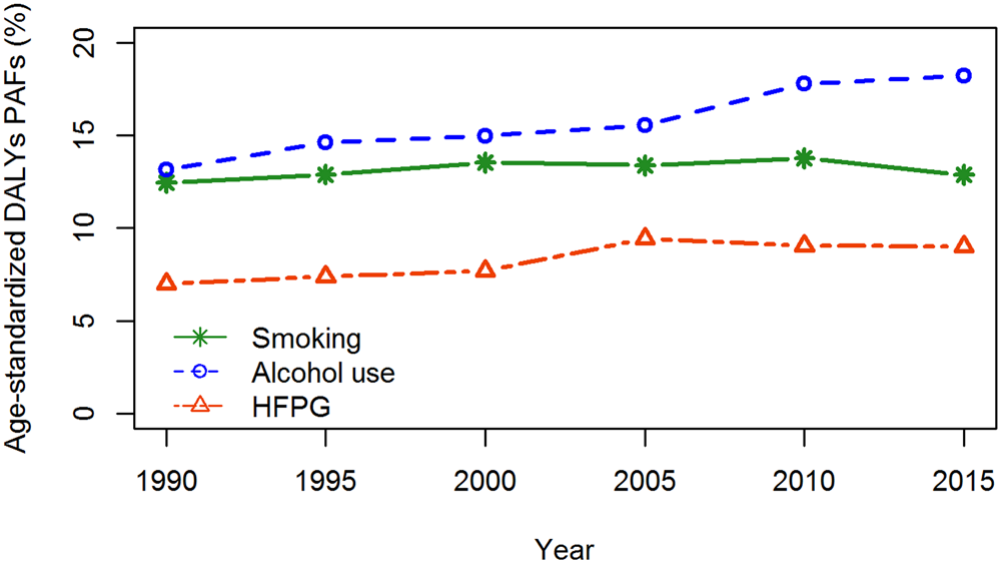
Changes in the age-standardized DALYs PAFs of TB from 1990 to 2015 for both sexes in China. Over the period 1990–2015, the attributable age-standardized DALY rates decreased for smoking, but increased for alcohol use and HFPG risk factors for both sexes.

In 2015, 33.82% (95% UI 28.14–39.83) of the all-age and 31.99% (95% UI 26.55–37.73) of the age-standardized DALY rates could be attributed to all risk factors of smoking, alcohol use, high fasting plasma glucose (HFPG) and their overlaps, whereas more than 66% could not be explicitly attributed to specific risk factors. The risk factor of alcohol use accounted for 17.17% (95% UI 14.61–19.64), followed by smoking (12.22% [95% UI 5.82–18.85]), and HFPG accounted for 8.35% (95% UI 5.30–11.88) of the attributable age-standardized DALY rates in 2015 for both sexes. Over the period 1990–2015, the attributable age-standardized DALY rates for smoking decreased by 12.98% (95% UI 2.40–24.27) for both sexes. However, an inverse pattern was observed for alcohol use and HFPG, which increased by 24.05% and 9.30%, respectively (Fig. 3).

The population attributable fraction (PAF) changes varied substantially in age- and sex-specific groups from 1990 to 2015 (Supplementary Table S2). The proportions of PAFs attributed to smoking decreased in all age groups, whereas a higher reduction was observed in females, with the highest reduction of 60.35% in the 50–69-year age group. Moreover, reductions in males increased as age increased. Unfortunately, the proportions of PAFs attributed to alcohol use and HFPG increased in all age groups; the highest increasing proportion in the 15–49-year age group due to alcohol use and HFPG surpassed 28% and 24%, respectively.

## Discussion

In this study, we mainly found marked reductions of nearly 70% in both the all-age or age-standardized mortality and DALYs from 1990 to 2015 in China for both sexes, but TB mortality and DALYs have faced a slow decline in recent years, which must be considered. The prevalence changed slightly for the all-age rate but decreased significantly for the age-standardized rate. Notable gender differences were observed: the prevalence steadily decreased in males from 1990 to 2015 but fluctuatingly changed in females with nearly increased by 6.24% in age-standardized prevalence of TB. Additionally, almost 30% of the age-standardized DALY rates were attributable to smoking, alcohol use and HFPG, consistently from 1990 to 2015 in China.

The highest mortality and DALYs have been steadily shifting to the elderly (70+ years of age) in both this study and global surveys^15^. The drift of TB to older populations has several causes. First, longevity is increasing in China. Life expectancy increased from 68.55 years in 1990 to 76.34 years in 2015^6^, and with longer life spans, more people survive to acquire TB infection. Second, the birth rate is decreasing. Reports from the National Bureau of Statistics of China indicate that birth rates have fallen from 21.06‰ in 1990 to 12.07‰ in 2015. Additionally, the entire population is aging. Thus, the proportion of the population older than 65 years was 5.60% in 1990 but increased to 10.50% in 2015^6^. In addition, health problems due to chronic disease or risk factors such as high total cholesterol, high systolic blood pressure, and hypoimmunity would predispose the elderly to TB^11,16,17^.

From the results of GBD 2015 and IHME, the subnational TB burden was not available. According to the GBD 2013, we found TB to be one of the top ten causes of years of life lost (YLLs) in Xinjiang and Tibet ranking 9^th^ and 10^th^, respectively, which ranked 4^th^ in 1990^18^. When making a comparison with top two TB burden countries, the age-standardized DALY rate per 100,000 persons in 2015 in China was 119.60 (95% UI 100.75–161.19), which was far lower than that observed in India [1427.61(95% UI 1145.12–1821.51)] and Indonesia [1462.29 (95% UI 1154.98–2028.64)]. Overall, compared with the worldwide rate and those for developed countries, the age-standardized DALY rate in China was far lower than that observed worldwide [552.42 (95% UI 467.18–681.30)] but far higher than that observed in high-income countries [14.82 (95% UI 13.98–15.70)] in 2015^4^. On the basis of these findings, it appears that China has made great progress in cultural status and health education compared with other developing countries, but DALYs has faced a slow decline in recent years, and further efforts are needed in the future.

The third national TB prevalence survey, which was implemented in 2010 and screened individuals aged older than 15 years, showed a reduced prevalence of TB for both sexes^19^. The GBD 2015 results indicated the same trend of TB prevalence in patients older than 15 years from 1990 to 2010 in both males and females in China. A reduced incidence, a high TB cure rate and a shortened TB duration may have resulted in the reduction of TB prevalence. Notably, the prevalence of TB increased since 2010 in females and in the populations aged under 5 and 5–14 years for both sexes^4^. Children are vaccinated with Bacille Calmette-Guerin (BCG), which prevents the dissemination of TB in young children and reduces the probability of infection TB by 50%^20^. Unfortunately, due to non-specific symptoms and the absence of an ideal diagnostic method, the prevalence of TB in children is often underestimated^21^.

The National Health and Family Planning Commission of the People’s Republic of China launched a nationwide scale-up policy on “school TB prevention and control work” in July 2010, which was reinforced by screening, reporting and prevention of new TB cases in kindergarten and all kinds of school students, including those in primary school, junior high school, senior high school and college^22^. In addition, a recent national youth tobacco survey in China indicated that 19.90% of students in junior high school used tobacco and that the rates of smoking in adolescent females under 15 years of age had increased. Furthermore, 72.90% of students had been exposed to second-hand smoking^23^. Active TB screening, early identification and a high rate of smoking may be the most important reasons for the increasing prevalence among children.

The prevalence of TB in females fluctuating changed and began to increase again in 2015 according to data from GBD 2015, which may be attributed to the following mechanisms. One reason was MDR-TB, which is a major threat to the control of TB^13^. In China, a national survey of drug-resistant TB indicated that the rate of MDR-TB among retreated and newly diagnosed TB cases was 25.60% and 5.70%, respectively. Additionally, a female predominance in MDR-TB risk among retreated or newly diagnosed TB patients was observed, with adjusted odds ratio of 2.20 (95% CI 1.40–3.50) and 1.50 (95% CI 1.00–2.10), respectively^24^. MDR-TB treatment is expensive, and patients must pay deductibles in China, which pose a heavy financial burden, particularly to those from poor households. MDR-TB may increase the percentage of patients who default from treatment, thus extending the overall treatment duration and increasing new TB transmission. Another reason may be a larger female population shift from rural to urban than previously, which can expose them to infection and lead to TB disease^6^. Apart from the above reasons and greater longevity, further national surveys to determine the latent reasons for the increasing prevalence in females are needed.

In addition to the limitations reported in GBD studies^7,25,26^, there are potential limitations in this study. First, it is difficult to capture the characteristics of TB sequela because symptoms such as cough, fever, short of breath and weight loss may be mild and thus ignored, which greatly affects YLD calculations^27^. Second, modelling the estimated risk factors of DALYs may not accurately capture how all risks interact. Apart from smoking, alcohol use and well-established HFPG risk factors, specific factors such as ambient air pollution, malnutrition and diabetes are known to increase both the risk of TB transmission and the development of active disease. Studies reported positive associations between particulate matter (PM_2.5,_ PM_10_), carbon monoxide (CO), nitrogen dioxide (NO_2_) and the development of TB in the general population^28,29^. The ambient air pollution PM_2.5_ has been a considerable public concern in China in recent years and is ranked as the 4^th^ greatest risk factor for disease burden, which should be systematically qualified using the available data^30^.

In summary, although we made great progress on TB control, the current epidemic situation of TB still remains serious in females especially in the under 5 years of age group. To meet global targets, more policies such as active case finding and preventive therapy in the latently infected should be implemented to control prevalence. The risk factors of smoking, alcohol use and HFPG were insufficient to explain TB DALYs and more latent reasons should be explored in future studies.

## Materials and Methods

### Data sources

National disease surveillance points, mortality registration and reporting system, national maternal and child health surveillance system and sample survey on population changes were main sources for China^3^. The TB cases were identified based on International Classification of Diseases and Injuries, versions 10 (ICD-10), discharge diagnosis code (codes A10-A14, A15-A19.9, B90-B90.9, K67.3, K93.0, M49.0, and P37.0). Risk factors, which are associated with an increased risk of DALYs, are potentially modifiable causes of disease or injury^26^. There were three main risk factors included in this study: tobacco smoking, excluding second-hand smoke; alcohol use; and HFPG. Nationally representative survey data, alcohol sales, epidemiological studies and examination surveys for HFPG are the sources of data for estimating TB risk factors. Because of the interdependency of HIV/AIDS and TB, the metrics of TB in this study were for people living without HIV. The datasets generated during analysed during the current study are available in the IHME, [http://ghdx.healthdata.org/gbd-results-tool].

### Statistical analysis

In order to comprehensively overview the TB trends and changes in mortality, prevalence and DALYs, we used Spatiotemporal Gaussian process regression to synthesize the sources and simultaneously correct for biases, and a causes-of-death ensemble modelling (CODEm) strategy to produce a complete dataset of exposure distribution and mortality^5,31^. Meanwhile, the non-fatal TB prevalence was estimated by using the Disease Model-Meta Regression (DisMod-MR 2.1)^25^. Thus, dynamical models, which can do both mathematical analysis and data fitting to reveal the transmission rules of infectious^32,33^, are not necessary in this paper. DALYs which represent how many years of healthy life are lost due to death and non-fatal illness or impairment are relatively simple and widely used to estimate the burden of disease. DALYs are equal to the sum of YLLs and YLDs^7^. YLLs assess the distance between observed mortality and a life expectancy and are computed by multiplying the number of TB deaths at each age χ by a standard life expectancy at age χ, whereas YLDs assess the health loss and are computed as the prevalence of TB sequelae multiplied by the disability weight. The sequelae of TB included a persistent cough and fever, shortness of breath, feelings weak, and substantial weight loss^12^. The standard life expectancy from the normative life table at the age of death was 86.59 years at the age of 0 year and 23.79 years at the age of 65 years, which was computed based on the lowest recorded death rates across countries^3^. The updated world population age standard, which was developed based on the GBD 2013 data sourced from the IHME website^34^, was redefined for the values of age-standardized mortality rates, prevalence and DALYs from 1990 to 2015.

The comparative risk assessment method is used to calculate the proportion of the TB burden that is attributable to risk factors^26^. Due to incomplete or missing data for risk factors for both specific populations and years, models such as Bayesian hierarchical regression, mixed effect regression and CODEm were used to generate a complete set of the current exposure distributions. Two formulas are presented to assess the PAF. The first is for polytomous tobacco smoking, and the second is for continuous alcohol use and HFPG^26^.

1 $${PAF}_{asy}=\frac{\sum _{x=1}^{n}{RR}_{asy}(x){P}_{asy}(x)-{RR}_{asy}({TMREL}_{asy})}{\sum _{x=1}^{n}{RR}_{asy}(x){P}_{asy}(x)}$$

Where ${PAF}_{asy}$ is the population attributable fraction for tobacco smoking for age group ${\rm{\alpha }}$, sex $s$ and year y. ${RR}_{asy}(x)$ is the relative risk (RR) at exposure level χ on a plausible range of exposure levels from 1 to n. ${RR}_{asy}({TMREL}_{asy})$ is the relative risk (RR) at theoretical minimum risk exposure level (TMREL). ${P}_{asy}(x)$ is the proportion of population in risk group.

2 $${PAF}_{asy}=\frac{{\int }_{x=m}^{n}{RR}_{asy}(x){P}_{asy}(x)dx-{RR}_{asy}({TMREL}_{asy})}{{\int }_{x=m}^{n}{RR}_{asy}(x){P}_{asy}(x)dx}$$

Where ${RR}_{asy}(x)$ is the relative risk (RR) at exposure level χ with the lowest level of observed exposure m to the highest n.

## Electronic supplementary material

Supplementary Table S1

Supplementary Table S2
