# Publisher Correction: The burden and challenges of tuberculosis in China: findings from the Global Burden of Disease Study 2015

**DOI:** 10.1038/s41598-018-19650-1

**Published:** 2018-01-24

**Authors:** Sui Zhu, Lan Xia, Shicheng Yu, Saobing Chen, Juying Zhang

**Affiliations:** 1 Department of Epidemiology and Biostatistics, West China School of Public Health, Sichuan University, Sichuan, 610044 China; 2 Sichuan Provincial Center for Disease Control and Prevention, Chengdu, P.R. China; 3 Office of Epidemiology, Chinese Center for Disease Control and Prevention, Beijing, P.R. China

Correction to: *Scientific Reports* 10.1038/s41598-017-15024-1, published online 03 November 2017

The original HTML version of this Article contained a typographical error in the publication date ‘03 November 2017’ which was incorrectly given as ‘06 November 2017’. This has now been corrected in the HTML version of the Article.

