# Supplementary material for: The burden and challenges of tuberculosis in China: findings from the Global Burden of Disease Study 2015
Source: Sci Rep. 2017 Nov 3;7:14601. doi: 10.1038/s41598-017-15024-1 (PMC5668247; doi:10.1038/s41598-017-15024-1)
Supplement: Supplementary file 1 — Supplementary Table S1 [file 41598_2017_15024_MOESM1_ESM.pdf]

The burden and challenges of tuberculosis in China: findings from the Global Burden of Disease Study 2015

Sui Zhu[1], Lan Xia[2], Shicheng Yu[3], Saobing Chen[1], Juying Zhang[1*]

[1] Department of Epidemiology and Biostatistics, West China School of Public Health, Sichuan University, Sichuan 610044, China;

[2] Sichuan Provincial Center for Disease Control and Prevention, Chengdu, P.R. China;

[3] Office of Epidemiology, Chinese Center for Disease Control and Prevention, Beijing, P.R. China.

Supplementary Table S1. TB mortality by age-sex from 1990 to 2015 in China (per 100,000 persons, 95% UI).

Age-sex	1990	1995	2000	2005	2010	2015	%Δ (1990-2015)
**Both Sexes**							
under 5	2.61(1.43-3.56)	1.62(1.07-2.32)	1.31(0.83-1.65)	0.75(0.49-0.92)	0.36(0.27-0.54)	0.22(0.16-0.36)	-91.66
5-14 years	1.28(0.89-1.54)	0.82(0.61-1.04)	0.61(0.46-0.77)	0.36(0.28-0.44)	0.27(0.21-0.35)	0.16(0.13-0.21)	-87.71
15-49 years	6.87(5.27-8.21)	5.51(4.35-6.92)	3.92(3.10-4.93)	2.68(2.08-3.43)	1.75(1.46-2.42)	1.26(1.05-1.8)	-81.59
50-69 years	42.46(29.2-52.11)	29.27(21.71-37.29)	20.44(16.08-26.24)	13.58(10.87-18.31)	8.34(7.26-12.64)	6.24(5.18-9.78)	-85.3
70+ years	128.55(87.74-171.57)	93.99(71.01-124.85)	69.05(55.28-91.77)	49.78(41.4-68.71)	32.9(28.49-51.86)	24.21(20.08-39.77)	-81.17
All ages	13.82(9.99-17.02)	10.44(8.01-13.02)	8.08(6.45-10.36)	6.31(5.15-8.47)	4.43(3.87-6.62)	3.54(2.97-5.52)	-74.42
Age-standardized	20.96(14.81-26.26)	14.86(11.4-18.79)	10.62(8.48-13.71)	7.48(6.19-10.14)	4.74(4.17-7.18)	3.4(2.86-5.37)	-83.79
**Male**							
under 5	3.74(2.16-5.92)	2.46(1.63-3.82)	2.15(1.28-2.71)	1.24(0.75-1.55)	0.56(0.41-0.91)	0.33(0.24-0.61)	-91.22
5-14 years	1.24(0.77-1.65)	0.84(0.54-1.18)	0.69(0.42-0.93)	0.38(0.25-0.51)	0.27(0.19-0.39)	0.16(0.12-0.24)	-87.34
15-49 years	7.89(5.38-10.91)	6.57(4.81-9.19)	4.88(3.51-6.89)	3.49(2.47-4.94)	2.39(1.79-3.55)	1.78(1.36-2.76)	-77.39

50-69 years	56.06(35.49-77.13)	39.41(26.40-55.03)	27.64(19.64-39.81)	18.85(13.88-27.09)	12.19(9.96-19.35)	9.36(7.62-15.49)	-83.31
70+ years	178.90(119.7-266.36)	130.07(91.03-196.69)	97.67(70.88-142.78)	72.51(53.32-108.36)	49.42(39.54-80.88)	36.81(29.73-62.41)	-79.43
All ages	17.13(11.35-24.17)	13.16(9.30-18.84)	10.41(7.54-15.10)	8.42(6.21-12.03)	6.18(4.90-9.75)	5.04(4.06-8.19)	-70.55
Age-standardized	28.06(18.47-40.85)	20.01(14.16-29.11)	14.58(10.59-20.91)	10.62(7.83-15.53)	7.01(5.61-11.19)	5.12(4.16-8.33)	-81.76
**Female**							
under 5	1.39(0.46-1.92)	0.68(0.35-0.86)	0.35(0.27-0.50)	0.19(0.14-0.25)	0.13(0.08-0.16)	0.09(0.05-0.12)	-93.48
5-14 years	1.33(0.90-1.64)	0.79(0.58-1.04)	0.52(0.4-0.72)	0.34(0.26-0.44)	0.27(0.20-0.34)	0.16(0.11-0.21)	-88.08
15-49 years	5.79(3.86-6.89)	4.40(3.20-5.34)	2.92(2.21-3.53)	1.83(1.39-2.23)	1.07(0.85-1.42)	0.71(0.57-1.03)	-87.75
50-69 years	27.74(17.08-33.14)	18.27(12.25-21.20)	12.8(8.87-15.04)	8.06(6.15-10.24)	4.36(3.65-6.69)	3.06(2.36-4.99)	-88.98
70+ years	88.77(52.68-114.22)	64.79(42.04-79.99)	45.26(33.13-57.75)	30.2(25.59-42.5)	18.3(15.22-30.87)	12.99(9.64-22.86)	-85.37
All ages	10.34(6.98-12.02)	7.57(5.40-8.81)	5.62(4.25-6.81)	4.07(3.33-5.35)	2.57(2.20-4.00)	1.94(1.51-3.15)	-81.28
Age-standardized	14.73(9.64-17.42)	10.18(7.12-11.89)	7.00(5.22-8.47)	4.59(3.78-6.08)	2.64(2.25-4.17)	1.79(1.39-2.92)	-87.84

TB: tuberculosis; UI: uncertainty intervals; %Δ : the percent changes of death rate from 1990 to 2015.
