# Supplementary material for: The burden and challenges of tuberculosis in China: findings from the Global Burden of Disease Study 2015
Source: Sci Rep. 2017 Nov 3;7:14601. doi: 10.1038/s41598-017-15024-1 (PMC5668247; doi:10.1038/s41598-017-15024-1)
Supplement: Supplementary file 2 — Supplementary Table S2 [file 41598_2017_15024_MOESM2_ESM.pdf]

The burden and challenges of tuberculosis in China: findings from the Global Burden of Disease Study 2015

Sui Zhu[1], Lan Xia[2], Shicheng Yu[3], Saobing Chen[1], Juying Zhang[1*]

[1] Department of Epidemiology and Biostatistics, West China School of Public Health, Sichuan University, Sichuan 610044, China;

[2] Sichuan Provincial Center for Disease Control and Prevention, Chengdu, P.R. China;

[3] Office of Epidemiology, Chinese Center for Disease Control and Prevention, Beijing, P.R. China.

Supplementary Table S2. Varied changes of PAFs of three risk factors in age-sex-specific from 1990 to 2015 in China.

Age	Sex	Smoking		Alcohol use		HFPG	
		Change (%)	95% UI	Change (%)	95% UI	Change (%)	95% UI
15-49 years	Male	-4.58	-11.39 to 3.46	30.16	20.17 to 38.38	37.08	28.91 to 48.9
	Female	-34.89	-52.77 to -9.99	28.1	9.74 to 47.87	24.12	12.42 to 46.01
	**Both**	1.97	-11.1 to 19.84	38.87	21.33 to 55.82	35.78	26.43 to 52.79
50-69 years	Male	-12.3	-15.58 to -9.86	14.35	6.29 to 23.48	10.09	5.53 to 14.85
	Female	-60.35	-68.84 to -50.68	25.61	7.65 to 43.99	5.97	-0.34 to 11.83
	Both	-10.88	-21.33 to -3.77	20.54	6.33 to 32.5	8.9	4.28 to 13.63
70+ years	Male	-17.45	-21.96 to -13.67	15.22	0.97 to 29.1	8.42	0.57 to 29.16
	Female	-21.69	-38.36 to 1.64	16.56	0.48 to 34.73	10.54	2.61 to 24.99
	**Both**	-11.73	-22.82 to -3.42	23.94	6.33 to42.01	8.59	1.24 to 26.38
All ages	Male	-1.59	-7.08 to 7.09	29.71	19.89 to 38.27	29.19	22.19 to 40.95
	Female	-34.91	-47.05 to -13.36	34.13	15.82 to 52.83	24.28	13.98 to 46.37
	Both	3.33	-10.73 to 17.85	38.38	19.9 to 53.04	28.98	21.35 to 44.13

Age-standardized	Male	-14.59	-18.81 to -8.65	19.52	9.69 to 28.43	13.11	7.56 to 20.85
	Female	-48.12	-57.71 to -31.59	20.58	5.05 to 38.01	-0.09	-7.76 to 14.8
	**Both**	-12.98	-24.27 to -2.41	24.05	7.90 to 37.32	9.3	3.40 to 19.53

PAFs: population attributable fractions; UI: uncertainty intervals; HFPG: high fasting plasma glucose.
